# Purely Off-Clamp Sutureless Robotic Partial Nephrectomy for Novice Robotic Surgeons: A Multi-Institutional Propensity Score-Matched Analysis

**DOI:** 10.3390/jcm13123553

**Published:** 2024-06-18

**Authors:** Cosimo De Nunzio, Giorgia Tema, Aldo Brassetti, Umberto Anceschi, Alfredo Maria Bove, Simone D’Annunzio, Mariaconsiglia Ferriero, Riccardo Mastroianni, Leonardo Misuraca, Salvatore Guaglianone, Gabriele Tuderti, Costantino Leonardo, Riccardo Lombardo, Antonio Cicione, Antonio Franco, Eugenio Bologna, Leslie Claire Licari, Sara Riolo, Rocco Simone Flammia, Antonio Nacchia, Alberto Trucchi, Giorgio Franco, Andrea Tubaro, Giuseppe Simone

**Affiliations:** 1 Department of Urology, Ospedale Sant’Andrea, Sant’Andrea Hospital, La Sapienza University, 00185 Rome, Italy; giorgiat88@hotmail.it (G.T.); rlombardo@me.com (R.L.); antonio.cicione@uniroma1.it (A.C.); anto.franco@hotmail.it (A.F.); sarariolomail@gmail.com (S.R.); antonio.nacchia@uniroma1.it (A.N.); alberto.trucchi@uniroma1.it (A.T.); andrea.tubaro@uniroma1.it (A.T.); 2 Department of Urology, IRCCS “Regina Elena” National Cancer Institute, 00144 Rome, Italy; aldo.brassetti@gmail.com (A.B.); umberto.anceschi@ifo.it (U.A.); alfredo.bove@ifo.it (A.M.B.); simone.dannunzio@ifo.it (S.D.); mariaconsiglia.ferriero@ifo.it (M.F.); riccardo.mastroianni@ifo.it (R.M.); leonardo.misuraca@ifo.it (L.M.); salvatore.guaglianone@ifo.it (S.G.); gabriele.tuderti@ifo.it (G.T.); costantino.leonardo@ifo.it (C.L.); roccosimone.flammia@ifo.it (R.S.F.); puldet@gmail.com (G.S.); 3 Urology Unit, Department of Maternal-Child and Urological Sciences, Policlinico Umberto I Hospital, “Sapienza” University of Rome, 00185 Rome, Italy; eugenio.bologna@uniroma1.it (E.B.); leslieclaire.licari@uniroma1.it (L.C.L.); giorgio.franco@uniroma1.it (G.F.)

**Keywords:** RAPN, robotic surgery, enucleation, kidney cancer, learning curve

## Abstract

**Objectives:** To compare perioperative outcomes of patients treated with sutureless off-clamp robotic partial nephrectomy (sl-oc RAPN) by either a novice or an expert robotic surgeon at two different institutions. **Methods:** Data concerning two continuous series of patients with cT1-2N0M0 renal tumors treated with sl-oc RAPN either by a novice or an expert surgeon were extracted from prospectively populated institutional databases over the last 4 years. Perioperative outcomes as well as the baseline characteristics of patients and tumors were compared by using χ^2^ and Mann–Whitney tests for categorical and continuous variables, respectively. A 1:1 propensity match score analysis (PMSa) generated two homogeneous cohorts. Logistic regression analysis was performed to assess predictors of trifecta outcomes, defined as negative surgical margins, no Clavien–Dindo ≧ 3 grade complications, and no ≧ 30% postoperative eGFR reduction. **Results:** Overall, 328 patients were treated by an expert surgeon, while 40 were treated by a novice surgeon. After PMSa analysis, two cohorts of 23 patients each were generated, homogeneous for all baseline variables (*p* ≥ 0.07). Hospital stay was the only significantly different outcome observed between the two groups (5 days vs. 2 days; *p* < 0.001). No statistically significant differences were recorded when comparing trifecta outcomes (expert: 100% vs. novice: 87%; *p* = 0.07). In the logistic regression analysis, no statistically significant predictors of trifecta outcomes were recorded. **Conclusions:** sl-oc RAPN is a feasible and safe nephron sparing technique, even when performed by a novice robotic surgeon.

## 1. Introduction

Partial nephrectomy (PN) is considered to be a common treatment option for renal cell carcinoma (RCC), with efforts focused on reducing invasiveness and preserving kidney function [1,2]. Robot-assisted PN (RAPN) has gained popularity due to its favorable perioperative and functional outcomes compared to open and laparoscopic techniques [3,4]. More specific techniques such as selective clamping and off-clamp RAPN have been developed to minimize ischemic injury and renal dysfunction [5,6]. Sutureless renorrhaphy has also emerged as a potential alternative to standard suturing, resulting in shorter operative times and improved perioperative outcomes [7]. Furthermore, the possibility to avoid isolating the renal hilum can prevent serious intraoperative complications, such as vessel or ureter injuries [5,6]. Indeed, the debate regarding whether or not to clamp the hilum persists and, despite the extensive literature available, definitive conclusions remain elusive. Antonelli et al. examined the safety profiles of different approaches by analyzing data from the first randomized trial ever conducted on the subject (CLOCK trial; NCT02287987) [8]. Furthermore, superselective clamping also did not provide better renal function preservation compared to renal artery clamping, questioning the benefit of this technique at a higher risk of intraoperative bleeding [9]. At the moment, ongoing research aims to improve long-term functional outcomes and minimize the risk of acute kidney injury [10]. Comparative studies have been conducted to assess the feasibility and safety of sutureless and off-clamp RAPN (sl-oc RAPN) [7]. In this setting, the aim of this study is to compare the perioperative outcomes of patients treated with sl-oc RAPN in two different institutions. More specifically, we want to assess the feasibility and safety of this challenging procedure in the hands of a novice robotic surgeon with no previous experience of kidney robotic surgery.

## 2. Materials and Methods

Data concerning two continuous series of patients >18 yo with cT1- 2N0M0 renal tumors treated with sl-ocRAPN by a novice surgeon (no previous robotic experience) or an expert surgeon (>300 robotic procedures) in the last 4 years were extracted from two prospectively populated institutional databases. All patients provided their written informed consent. This study was conducted in accordance with the declaration of Helsinki and approved by the local ethics committee: IRU study—Prot. n. 258 SA_2021.

Detailed clinical history was collected, and physical examination was conducted in all patients. Age, gender, history of hypertension and diabetes, preop glomerular filtration rate (GFR), clinical stage, and ASA score were recorded. Data on tumor size, location, and R.E.N.A.L. score were recorded through a CT scan.

Postoperative outcomes included data on postoperative GFR, Hb drop ≥3.5 g/dl, length of hospital stay (LoS), and perioperative complications. Complications were graded according to the Clavien–Dindo (CD) classification system [11]. All surgical specimens were analyzed by a dedicated uropathologist. TNM and AJCC classifications were adopted for tumor staging and grading.

### 2.1. Surgical Technique

**Figure 1:**
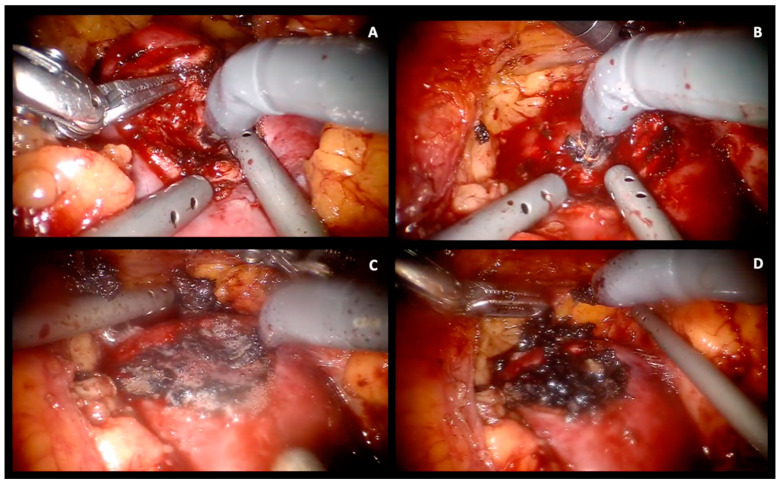
Intraoperative steps for sl-oc RAPN. (**A**) Pinpoint coagulation for bleeding vessels during enucleoresection. (**B**) Repeated forced monopolar coagulation on the tumor bed. (**C**) Eschar formation. Monopolar energy is administered in an almost direct contact manner, accompanied by gentle irrigation. (**D**) Two-minute time surgical field inspection after complete tumor bed coagulation.

Using a transperitoneal approach, a robotic trocar is inserted via open access para-umbilically to establish pneumoperitoneum. An endoscopic 30° camera is introduced, followed by the insertion of two 8 mm robotic trocars, one in the subcostal space and the other at the McBurney point. An AirSeal trocar and a 5 mm trocar are inserted 5 cm below and above the optic trocar, respectively (Supplementary Figure S1A). An intra-abdominal pressure of 12 mm Hg is used during the entire procedure. Told’s fascia is resected and the colon is medialized. Gerota’s fascia and the perirenal fat are opened according to the tumor site. When feasible, the adipose tissue overlying the tumor is preserved to ensure accurate pathological staging. The renal parenchyma around the tumor is marked using monopolar coagulation (Supplementary Figure S1B). Intraoperative ultrasonography is used to define the extent and depth of the renal mass, and to help mark the proposed excision line. For complex or endophytic tumors, indocyanine green may be utilized to assess the vascularization of the surrounding area. We have always performed a totally clampless tumor resection; the hilum has only been isolated in complex cases such as those totally endophytic or adjacent to the renal hilum masses. The tumor is excised using robotic scissors and is bluntly dissected. When bleeding vessels are visualized, the forced monopolar mode is utilized for pinpoint coagulation (Figure 1A). Once tumor excision is completed, repeated forced monopolar coagulation is performed on the tumor bed (Figure 1B) until a complete dry eschar is obtained. To avoid eschar adhesion to the monopolar scissor, energy is administered in an almost direct contact manner, accompanied by gentle irrigation (Figure 1C). If an incidental opening of the calyces happens, it is sutured using a 4/0 resorbable monofilament running suture. Following complete tumor bed coagulation, a two-minute time surgical field inspection is routinely performed and hemostasis is further checked (Figure 1D). A hemostatic agent (Floseal^®^) can be applied to the tumor bed. The excised mass is placed in a 10 mm EndoCatch retrieval bag (Ethicon, Sommerville, NJ, USA) and removed. Gerota’s fascia and the overlying peritoneum are closed with a running barbed suture. A drain is usually left in the renal fossa for at least 24 h.

### 2.2. Statistical Analysis

χ^2^ and Mann–Whitney tests were used for categorical and continuous variables, respectively. A 1:1 propensity match score analysis (PMSa) generated two cohorts homogeneous for demographics; ASA score; tumor size and complexity, graded according to RENAL score (categorical variable); and baseline estimated glomerular filtration rate (eGFR). Postoperative outcomes such as postoperative eGFR, hemoglobin loss, LoS, histology, and trifecta outcomes were recorded. Logistic regression analysis assessed predictors of trifecta outcomes, defined as negative surgical margins (NSM), no CD ≧ 3 grade complications, no ≧ 30% postoperative eGFR reduction (significant renal function deterioration; sFRD) [12].

## 3. Results

### 3.1. Overall Population

Overall, 368 patients with a median age of 62 (53/71) years were enrolled. Among them, 328 patients were treated by an expert surgeon while 40 were treated by a novice surgeon. All patients underwent a sl-oc RAPN (Table 1).

### 3.2. Expert vs. Novice Surgeon before PMSa

Before PMSa, tumors treated by the expert surgeon presented a lower rate of ASA scores > 2 (15% vs. 57%; *p* < 0.05), a higher rate of T2 cases (11% vs. 0%; *p* < 0.05), and a higher RENAL score (10 cases, 23% vs. 0%). In terms of postoperative outcomes, patients treated by a novice surgeon experienced longer LoS (5 d, IQR 5/6 vs. 2, IQR 2/3, *p* < 0.001), higher positive surgical margins (R1) (2% vs. 0%, *p* = 0.01), and higher eGFR reduction and Hb drop ≥ 3.5 g/dL, respectively, 84.6 (IQR 67.9/101.7) vs. 75.2 (IQR 59/90.6) mL/min/1.73 m^2^ (*p* = 0.01) and 2% vs. 0% (*p* = 0.01) (Table 2).

### 3.3. Expert vs. Novice Surgeon after PMSa

**Figure 2:**
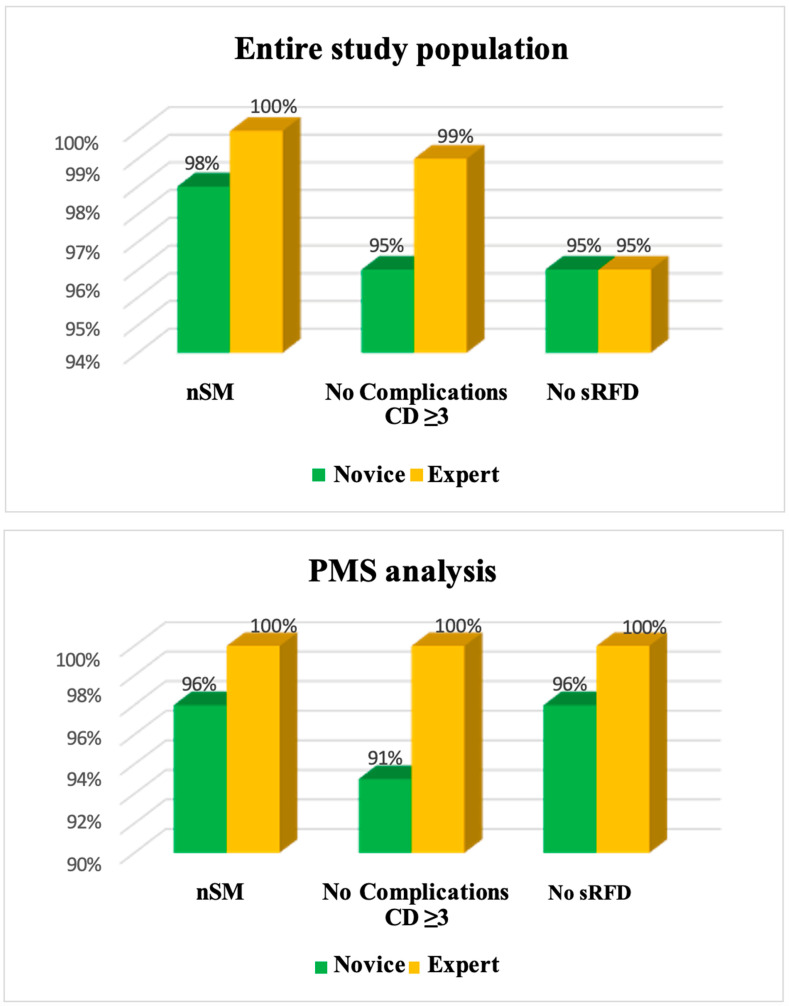
Surgical outcomes according to surgical experience. Abbreviations: nSM = negative surgical margins; sRFD = significant renal function deterioration; PMS = propensity score match.

After propensity matching, a total of 46 patients, two cohorts of 23 patients each, with a median age of 65 (53/72) years were compared. Overall, all baseline variables were homogenous among the two groups (Table 2). Regarding tumor features, no significant differences were observed between novice and expert surgeons in terms of tumor size (3.5 cm, IQR 2.5/4.5 vs. 4 cm, IQR 3/5, *p* = 0.44); clinical stage (0% vs. 13%, *p* = 0.07); and RENAL score (*p* = 0.23). Postoperative LoS was the only significantly different outcome observed (5 d, IQR 5/6 vs. 2, IQR 2/2, *p* <0.001). No statistically significant differences were recorded when comparing trifecta outcomes (expert: 100% vs. novice: 87%; *p* = 0.07). Likewise, no statistically significant differences were recorded in terms of R1 (expert: 0% vs. novice: 4%; *p* = 0.31), complications (expert: 0% vs. novice: 9%; *p* = 0.15), and renal function deterioration (expert: 0% vs. novice: 4%; *p* = 0.07) (Table 2) (Figure 2).

In the logistic regression analysis, no statistically significant predictors of trifecta achievement were recorded (Supplementary Table S1).

## 4. Discussion

In our study, we present the first experience with sl-oc RAPN performed by a novice robotic surgeon or by a high-volume surgeon for cT1–2 renal masses. Overall, good functional and oncological outcomes can be achieved with this technique, even in the hand of a novice robotic surgeon. Indeed, in a recent study by Mottrie et al. [13], PN required a short learning curve (just 30 cases) to gain a warm ischemia time (WIT) of less than 20 min, a console time less than 100 min, limited blood loss, and acceptable complication rates. In our study, the novice robotic surgeon did not have any experience with the robot platform and succeeded in performing all the cases without the need of an expert robotic surgeon; however, it was highly skilled in laparoscopic PN.

In the ongoing debate regarding whether or not to clamp during PN, it is important to highlight that the issue remains controversial. While zero-ischemia procedures, off-clamp, and superselective clamping of accessory arteries feeding the tumor have been introduced to minimize ischemia, none of these approaches have conclusively demonstrated superior outcomes in terms of oncological control or preservation of renal function, compared to standard clamping procedure [8,9,14]. In fact, although off-clamp techniques aim to reduce or eliminate ischemia time, they are associated with a high risk of bleeding at the tumor bed, which can severely impair visibility during tumor enucleation and increase the likelihood of positive surgical margins. This, in turn, can lead to further complications and, in cases of significant hemorrhage, acute renal function deterioration. As a matter of fact, in the CLOCK trial, a shift to on-clamp procedure was observed in 40% of the cases [8].

On the other hand, evidence suggests that patients undergoing cold ischemia or zero-ischemia procedures generally experience minimal renal function impairment, while the use of prolonged warm ischemia has been linked to a greater risk of significant renal damage. In particular, although a 30 min WIT was historically considered to be the threshold for renal pedicle block and, as suggested by Rod et al., there was no difference between a WIT < 25 min and zero ischemia time [15], conversely, Thompson et al. believe that reducing WIT is significant for the preservation of kidney function. In fact, outcomes from their interesting study entitled “Every minute counts”, showed that each decrease of a minute of WIT facilitated the preservation of renal function [16]. Furthermore, the possibility to avoid isolating the renal hilum can prevent serious intraoperative complications, such as vessel or ureter injuries [5]. Notably, in the CLOCK trial, it was mandatory to isolate the hilum in both the on-clamp and off-clamp groups and, therefore, outcomes related to perioperative complications may be misleading, especially for novice surgeons, and may not reflect the potential benefit of the pure off-clamp technique avoiding major complications related to the isolation of the renal artery and vein [17].

Other factors may be responsible for renal function preservation [18,19,20]: Bahler et al. believe that the renal parenchyma’s reconstruction is the most crucial factor for the preservation of kidney function after PN [21], while Zabell et al. concluded that it is the volume and mass of renal parenchyma preserved that represent the major factor affecting renal function [20]. In conventional PN, two-layer sutures are usually required. The suturing process usually begins with the basal layer, primarily focusing on suturing the blood vessels and the collective system. Subsequently, in the second layer, the renal parenchyma is sutured [22]. Reducing the number of sutures whenever feasible is considered to be an important option for preserving renal function. Zhao et al. demonstrated that there is no clear WIT threshold that has a clear impact on renal function, and that the crucial determinant for kidney function is the amount and quality of preserved renal parenchyma [23]. Another systematic review by Bertolo et al. [24] found that single-layer suturing showed better outcomes compared to double suturing in terms of renal function, thus conferring better preservation of renal function. Recently, Jin et al. compared a safe and feasible sutureless PN technique to a standard suturing PN, showing that sutureless PN exhibited a lower WIT, lower acute kidney injury rate, but similar eGFR decline [25]. Moreover, they reported impressive outcomes in terms of operative time, perioperative complications, and renal function preservation, thus rendering this technique a further advancement in the surgical management of small renal masses.

In our series, the rate of renal function deterioration was consistent with previous reports, which have cited rates ranging from 76% to 96%. In terms of perioperative outcomes, only one patient (3.4%) in the novice surgeon group experienced intraoperative complications, namely venous bleeding. Similar results in terms of perioperative complication rates were obtained for the novice sl-oc RAPN (90%) and expert sl-oc RAPN (93%) groups, even when PMSa was performed (100% vs. 87%), in accordance with previous reports of PN series [26,27]. Furthermore, no technique-related complications, such as major bleeding or urinary fistulas, were recorded among the two cohorts of patients. This observation highlights the safety of performing sl-oc RAPN, even with a novice robotic surgeon, and suggests that cortical renorrhaphy may be safely omitted [7]. Indeed, the only distinguishing factor between the two groups was LoS, which was shorter for patients treated by expert surgeons compared to those treated by novice surgeons (2 vs. 5 d; *p* = <0.001). A possible explanation for this result could be that patients treated by the novice surgeon were monitored more carefully during their hospital stay, and that postoperative management protocols might differ between the two institutions.

However, trifecta outcomes between the two groups did not statistically differ, which is clearly a promising result. Indeed, we decided to adopt the novel composite trifecta outcomes proposed by Brassetti et al., which have the ability to predict both oncologic and functional endpoints of PN [12].

In general, as suggested by Simone et al., a ‘zero ischemia’ PN is possible and may overcome the ischemia issue, especially for renal tumors with a low nephrometry score [14]. In fact, the authors stated that this is a reasonable approach for small and peripheral tumors, and that the technique has a low complication rate and excellent functional outcomes, without impairing oncological results. In our study, we confirmed these findings, performing a pure sl-oc RAPN in all of our patients. To note, our analyses depended on propensity score matching methodology; hence, this approach reduced the impact of selection bias by accounting for patient and tumor differences between the two cohorts. Nonetheless, the median tumor size was 3.5/4 cm, with only 13% being cT2 tumors (in the expert cohort), and all masses were categorized as having a RENAL score < 10. Certainly, this factor may bias our results, but it is also reasonable for an unexperienced surgeon to start with simple/intermediate cases. Actually, Ferriero et al. recently assessed the safety and feasibility, as well as oncologic and functional outcomes, of sl-oc RAPN for purely hilar renal masses (median RENAL score ≥ 10) in a single, high-volume center experience. Overall, no major complications were encountered and only one patient needed a blood transfusion [28]. Therefore, this technique is possible, even in more complex cases, maintaining optimal results.

Taken together, our findings demonstrate the safety and feasibility of sl-oc RAPN, even when performed by a novice surgeon. However, it is important to acknowledge some limitations of our study. The major limitations of the study include the small sample size, and our results may therefore be impacted by the enrolled population. Furthermore, we certainly recognize that it takes more than one study and one cohort of patients to prove a hypothesis, and therefore we hope that further comparative analyses will seek to report the outcomes of different PN techniques, understanding the advantages and drawbacks and their related learning curves. Another limitation is our reliance on retrospective data, and our study is subject to all the limitations associated with a retrospective study design. Finally, the current study does not provide information regarding operative and console times, which have a well-known impact on surgical procedure. Notwithstanding all these limitations, to the best of our knowledge, this is the first study comparing a new technique performed by a novice or an expert surgeon. We gained promising results for trifecta and perioperative outcomes, but prospective randomized trials are warranted to further validate the impact of sutureless and off-clamp techniques on long- term functional outcomes.

## 5. Conclusions

sl-oc RAPN is a feasible and safe nephron sparing technique, even when performed by a novice robotic surgeon. Intraoperative and postoperative complication rates are in line with the literature reports. Further studies evaluating the impact of sutureless and off-clamp techniques are needed to better understand the long-term functional outcomes.

## Figures and Tables

**Table 1:** Baseline characteristics and main outcomes of overall cohort.

Sl-oc RAPN (*n* = 368)	
Age, yrs	62 (53/71)
Gender, *n* (%)	
Male	207 (63%)
Female	121 (37%)
ASA score, *n* (%)	
≥3	73 (20%)
≤3	295 (80%)
Preop-eGFR, mL/min/1.73 m^2^	83 (69.2/97.3)
Clinical Tumor size, mm	3.5 (2.5/5)
cT2 stage, *n* (%)	37 (10%)
RENAL score, *n* (%)	
≤6	147 (40%)
7–9	147 (40%)
≥10	74 (20%)
Postop-eGFR, mL/min/1.73 m^2^	76.4 (60.3/91.7)
Hb drop ≥ 3.5 g/dL, *n* (%)	1 (0.5%)
LOS, days	2 (2/3)
Benign histology, *n* (%)	120 (33%)
Trifecta	342 (93%)
R1, *n* (%)	1 (0.5%)
Complications CD ≥ 3, *n* (%)	7 (2%)
sRFD, *n* (%)	20 (5%)

Data were reported in median/IQR. Abbreviations: sl-oc RAPN = sutureless off-clamp robot-assisted partial nephrectomy; ASA = American Society of Anesthesiologists; Preop-eGFR = preoperative estimated glomerular filtration rate; LOS = length of stay; R1 = positive surgical margins; CD = Clavien–Dindo complication scale; sRFD = significant renal function deterioration.

**Table 2:** Pre- and post propensity match score analysis according to surgeon experience.

	PRE PMSa			POST PMSa		
	Novice (*n* = 40)	Expert (*n* = 328)	*p*	Novice (*n* = 23)	Expert (*n* = 23)	*p*
**PREOPERATIVE CHARACTERISTICS**						
Age, yrs	68 (55/73)	61 (53/71)	0.06	68 (53/72)	62 (52/72)	**0.50**
Male gender, *n* (%)	22 (55%)	211 (64%)	0.25	14 (61%)	13 (56%)	**0.76**
ASA score ≥ 3, *n* (%)	23 (57%)	50 (15%)	**<0.001**	7 (30%)	9 (39%)	**0.54**
Preop-eGFR, mL/min/1.73 m^2^	82.9 (69.9/98.9)	82.9 (68.8/97)	0.16	90.3 (73.5/99.1)	81.4 (63.8/92.9)	**0.18**
Clinical tumor size, mm	3 (2.5/4.5)	3.5 (2.5/5)	0.48	3.5 (2.5/4.5)	4 (3/5)	**0.44**
cT2 stage, *n* (%)	0 (0%)	37 (11%)	**0.02**	0 (0%)	3 (13%)	**0.07**
RENAL score, *n* (%)			**0.001**			**0.23**
≤6	24 (60%)	123 (37%)		11 (48%)	13 (56%)	
7–9	16 (40%)	131 (40%)		12 (52%)	10 (44%)	
≥10	0 (0%)	74 (23%)		0 (0%)	0 (0%)	
**POSTOPERATIVE OUTCOMES**						
Postop-eGFR, mL/min/1.73 m^2^	84.6 (67.9/101.7)	75.2 (59/90.6)	**0.01**	84.6 (81.2/102.2)	81.7 (66.6/88.4)	**0.06**
Hb drop ≥ 3.5 g/dL, *n* (%)	1 (2%)	0 (0%)	**0.01**	1 (4%)	0 (0%)	**0.31**
LOS, d	5 (5/6)	2 (2/3)	**<0.001**	5 (5/6)	2 (2/2)	**<0.001**
Benign histology, *n* (%)	12 (30%)	108 (33%)	0.71	7 (30%)	11 (48%)	**0.23**
Trifecta	36 (90%)	306 (93%)	0.44	20 (87%)	23 (100%)	**0.07**
R1, *n* (%)	1 (2%)	0 (0%)	**0.01**	1 (4%)	0 (0%)	**0.31**
Complications CD ≥ 3, *n* (%)	2 (5%)	5 (1%)	**0.13**	2 (9%)	0 (0%)	**0.15**
sRFD, *n* (%)	2 (5%)	18 (5%)	**0.90**	1 (4%)	0 (0%)	**0.31**

Data are reported as median (IQR). Abbreviations: PMSa = propensity match score analysis; ASA = American Society of Anesthesiologists; Preop-eGFR = preoperative estimated glomerular filtration rate; Postop-eGFR = postoperative estimated glomerular filtration rate; LOS = length of stay; R1 = positive surgical margins; CD = Clavien–Dindo complication scale; sRFD = significant renal function deterioration.

## Data Availability

The data presented in this study are available on request from the corresponding author due to privacy policies.

## Supplementary Materials

The following supporting information can be downloaded at: https://www.mdpi.com/article/10.3390/jcm13123553/s1, Figure S1: (A) Ports placement for sl-oc RAPN. (B) Defining tumor margins using monopolar coagulation. Legend: Blu circle: 8 mm Robotic Port; Yellow circle: 8 mm Camera Port; Red Circle: 12 mm Airseal Port, 5 mm Assistant port. Table S1: Logistic regression analysis for the prediction of Trifecta.
