# Supplementary material for: Purely Off-Clamp Sutureless Robotic Partial Nephrectomy for Novice Robotic Surgeons: A Multi-Institutional Propensity Score-Matched Analysis
Source: J Clin Med. 2024 Jun 18;13(12):3553. doi: 10.3390/jcm13123553 (PMC11204664; doi:10.3390/jcm13123553)
Supplement: Supplementary file 1 [file jcm-13-03553-s001.zip › jcm-3051726-supplementary.pdf]

Supplementary Material

Table S1. Logistic regression analysis for the prediction of Trifecta.

	Univariable analysis				Multivariable analysis			
		95% CI				95% CI		
	OR	Lower	Higher	p	OR	Lower	Higher	p
Age	1.07	0.96	1.18	0.23	-	-	-	-
Male gender	0.69	0.06	8.26	0.77	-	-	-	-
ASA score ≥3	1.07	0.09	12.8	0.96	-	-	-	-
Preop-eGFR	0.95	0.88	1.02	0.15	-	-	-	-
Clinical tumor size	0.98	0.46	2.05	0.95	-	-	-	-
RENAL score ≥ 7	0.43	0.04	5.16	0.51	-	-	-	-
Extracapsular Ext.	1.61	0.00	-	0.99	-	-	-	-

Abbreviations: ASA = American Society of Anesthesiologists, Preop-eGFR = preoperative estimated glomerular filtration rate, Ext = Extension.

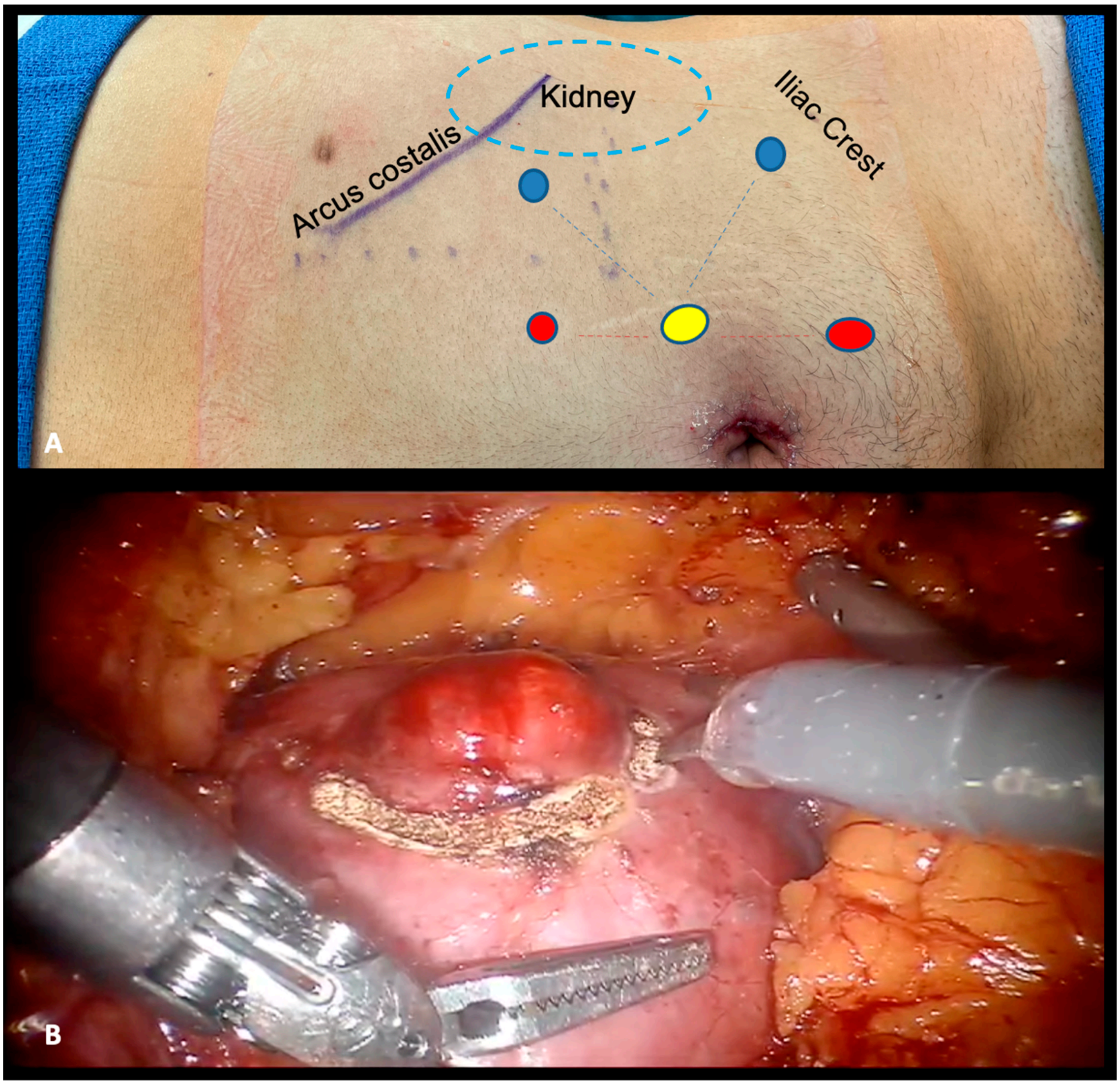

Figure S1. (A) Ports placement for sl-oc RAPN. (B) Defining tumor margins using monopolar coagulation. Legend: Blu circle: 8 mm Robotic Port; Yellow circle: 8 mm Camera Port; Red Circle: 12 mm Airseal Port, 5 mm Assistant port.
